# Epidemiologic analysis of breast cancer incidence, prevalence, and mortality in India: Protocol for a systematic review and meta-analyses

**DOI:** 10.1097/MD.0000000000013680

**Published:** 2018-12-28

**Authors:** Madurantakam Royam Madhav, Sankaranarayanan Gomathi Nayagam, Krishnav Biyani, Venkatesh Pandey, Duraj Gowtham Kamal, Shanthi Sabarimurugan, Nachimuthu Ramesh, Kodiveri Muthukaliannan Gothandam, Rama Jayaraj

**Affiliations:** a Department of Bio-Medical Sciences, School of BioScience and Technology, Vellore Institute of Technology (VIT) University, Vellore, India; b College of Health and Human Sciences, Charles Darwin University, Darwin, Australia.

**Keywords:** breast cancer, epidemiology, incidence, India, meta-analyses, mortality, prevalence, protocol, systematic review

## Abstract

**Background:**

Breast Cancer (BC) is the leading cause of deaths in Indian women. Emerging reports reveal alarming evidence of increasing incidence and mortality of BC among young Indian women in addition to the late presentation and poor prognosis. Despite the significant incidence, there is a lack of reliable data resources and comprehensive epidemiologic studies relating to BC. The objective of this protocol is to conduct a full-scale systematic review and meta-analyses on the incidence, prevalence, and mortality of BC in 29 states and seven union territories of India.

**Methods:**

Data sources used will be Cochrane Review, MEDLINE, PubMed, Scopus, Science Direct, Web of Science, and international and national cancer registries such as World Health Organization, International Agency for Research on Cancer (IARC), and National Centre for Disease Information and Research (NCDIR)-National Cancer Registry Program initiated by Indian Council of Medical Research. Relevant data will be extracted using a predefined data collection form. A defined search strategy will be implemented along with selection criteria to obtain full-text articles of relevant studies. This study protocol was prepared according to the Preferred Reporting Items for Systematic Reviews and Meta-Analysis for Protocols 2015 guidelines. Odds ratios (ORs) will be used to measure effect size. The random or fixed-effects meta-analyses model will be employed to aggregate the pooled estimates (ORs) with 95% confidence intervals (CIs) separately. A forest plot will be produced to assess ORs and 95% CIs. Publication bias will be assessed using funnel plot, and Egger regression will be applied to test the symmetry of the funnel plot.

**Ethics and dissemination:**

This proposed study will be based on published studies and the data from cancer registries. Therefore, human research ethics approval is not required. The results of this study will be published in a peer-reviewed journal.

**PROSPERO registration no:**

CRD42018084003.

## Abstract 2

Supplemental Digital Content is available in the text

## 1 Introduction

### 1.1 Epidemiology

Breast cancer (BC) is the most common incident sites of cancer in women worldwide.^[1,2]^ Asia has 44% of the world's BC deaths with 39% of overall new BC cases diagnosed.^[3]^ Approximately 25% of the female cancer cases in India are BC.^[4,5]^ The rate of incidence was found to be 25.8 in 100,000 women and the mortality rate is 12.7 per 100,000 women (2017).^[4]^ The highest rate of occurrence was found to be in Delhi (41 per 100,000 women) followed by Chennai (37.9 per 100,000 women), Bangalore (34.4 per 100,000 women), and Thiruvananthapuram district (33.7 per 100,000 women).^[4]^ The mortality-to-incidence ratio, when analyzed, was found to reach 0.66 in rural registries and 0.08 in urban registries.^[4]^ Another troubling concern about the scenario of BC in India is the increased incidence of the disease in younger Indian women (between the ages of 30 and 40).^[4,5]^ Presently, almost 48% of patients with BC in India are below 50 years of age. There is an increasing trend of BC in women between the ages of 25 and 40 in the past 25 years.^[5]^ The latest surveillance trends from 2000 to 2014 based on registries from 71 countries estimated the 5-year survival ratio to be 66.1% in India which is the lowest levels among the countries included in their study.^[6]^

### 1.2 Rationale

#### 1.2.1 The importance of the issue

The primary issue in estimating the incidence, prevalence, and mortality of BC in Indian scenario is the delayed hospital presentation and diagnosis, scarcity of hospital-based databases and electronic cancer registries, and state and nation-wide interconnected registration practices.^[4]^ The majority of data on BC incidence comes from local studies in a specific geographical location and source for a less number of cities.^[5]^ The recently updated National Institute of Cancer Registry Programme reports state a need to establish the accurate epidemiology of BC.^[5]^ Even though there is a review covering the prevalence of triple-negative BC(TNBC) in India, there is no systematic reviews and meta-analyses that have evaluated the age-standardized incidence, 1-, 3-, and 5-year prevalence and age-standardized mortality of BC in 29 states and seven union territories of India. Also, emerging studies show that there is sufficient data available to perform systematic review and meta-analysis on the epidemiology of Indian patients with BC.^[7]^

A preliminary search of PROSPERO, MEDLINE, and the Cochrane Database of Systematic Reviews were conducted, and no current or underway systematic reviews on this topic were identified.

### 1.3 How will the study address this issue?

This proposed study has a potential to develop a broad picture of BC epidemiology from all the existing literature on relevant published studies and databases in the Indian subcontinent. The study builds on the knowledge gained from the qualitative and quantitative data for incidence, prevalence and mortality in the Indian population in addition to age-wise BC trends and projection of burden of BC. The key findings of the proposed study will address the improvement in future study design, identify the inherent drawbacks, and assist in eliminating them.

### 1.4 How will it help?

This study will help in understanding the specific differences among innumerable factors such as diverse demographic, clinicoepidemiologic, clinicopathologic, and biologic characteristics of Indian patients with BC. The output of the systematic review and meta-analysis will generate new hypotheses and is likely to be useable synergistically. Our findings will aid in the identification of high-risk BC population, developing guidelines for early screening and management and creating awareness among the Indian women.

### 1.5 Review questions

The objective of our systematic review protocol is to describe the methodologic approach for conducting a systematic review and meta-analysis to explore the incidence, prevalence, and mortality rate in BC in India.

1. To conduct the full-scale systematic review and meta-analysis on the incidence, prevalence, mortality of BC in 29 states and 7 union territories of India.
2. To study the prevalence, incidence and mortality rate in age-wise BC trends and projection of burden of BC.
3. To study the diverse demographical, clinicoepidemiologic, clinicopathologic, and biologic characteristics of Indian patients with BC.

### 1.6 Participants

Inclusion criteria:

- Participants of all ages with a clear indication about the studies with participant age and its range.
- Participants with test-based confirmation of BC.
- Participants are residing in 29 states and seven union territories of India.

Exclusion criteria:

- Self-reporting, employing the screening methods of deduction and questionable survey.

### 1.7 Selection criteria for studies

Inclusion criteria:

- Studies that reported BC metrics for women residing in India
- Studies that included where a BC diagnosis was based on a histologic review
- Studies carried out with independent data collection
- Studies are providing statistical figures regarding the epidemiology of BC in India
- Studies are describing the geographical site
- English language publication

Exclusion criteria:

- Studies that clearly state the data being commenced upon is not original and has been taken from a cancer registry (either of national or global origin)
- Studies carried out as a narrative review
- Studies that have been duplicated

### 1.8 Outcomes and prioritization

#### 1.8.1 Primary outcomes

The primary outcome is to investigate the age-standardized incidence; 1-, 3-, and 5-year prevalence, and age-standardized mortality of BC in India.

#### 1.8.2 Secondary outcomes

The secondary outcomes are to compare the variations in primary outcomes with different geographic locations in India in addition to other demographic, clinicoepidemiologic, clinicopathologic, and clinic parameters.

### 1.9 Types of studies

The study design would be epidemiologic reports, cohort studies, and individual studies that have reported the odds ratio and confidence intervals on patients’ survival and incidence will be considered. Also, studies which demonstrate other demographic, clinicopathologic parameters, and immunohistochemical detection results will be considered for subgroup analysis. Studies published from the database for the last 10 years to the present will be included.

## 2 Methods

The proposed systematic review will be conducted in accordance with the Preferred Reporting Items for Systematic Reviews and Meta-Analyses (PRISMA) guidelines.^[8]^ Studies will be extracted based on the following criteria: study design, participants, eligibility criteria for studies and participants, and setting.

### 2.1 Search strategy

A draft search strategy for the databases has been included to identify the studies describing the incidence, prevalence and mortality of BC in India. The search string will be used as shown in Supplemental Digital Content (Appendix 1). A comprehensive search strategy for published studies will be carried out using Medical Subjective Heading terms using the electronic databases; Cochrane Review, Embase, MEDLINE, PubMed, Scopus, Science Direct, and Web of Science published until June 2018. A manual search of the reference list of the included studies will be done to identify relevant studies, and final electronic search strategies will be defined by the corresponding author (RJ).

### 2.2 Searching other resources

We will also examine conference proceedings related to this topic. In addition, epidemiologic data from the following reports by cancer registries will be incorporated:

1. GLOBOCAN 2012 by World Health Organization (WHO): International Agency for Research on Cancer (IARC)
2. Cancer incidence in 5 Continents (CI5) by WHO: IARC
3. Global Cancer Observatory by WHO: IARC
4. Global Heath Estimate 2012 by WHO: Department of Health Statistics and Information Systems
5. Three-Year Report of Population-Based Cancer Registries 2012-2014 by National Centre for Disease Information and Research (NCDIR)-National Cancer Registry Program (NCRP) maintained by Indian Council of Medical Research (ICMR)

If we are not able to retrieve required and sufficient data, we will be considering other reports from NCRP, national mortality data from Civil Registration System, Medical Certification of Cause of Death (MCCD) and the Sample Registration System and Global Burden of Disease specifically Local Burden of Disease as well as·State-level disease burden initiative in India.

### 2.3 Study design and participants

The studies explaining the confirmative diagnosis by clinical examinations and conclusive test for incidence, prevalence and mortality of BC in India will be included. The studies conducted on general population will be included while no limits on study participants age, ethnicity, morbidity, and occupation will be implied. Studies that have been carried out independently without the influence of any of the international or national registry data on the epidemiology of BC will be added. The mortality data would be obtained from MCCD. The language of publication will be restricted to English while the publication date and status will be unrestricted. Standardized outcome measures of age, state, and city-wise distribution of incidence, prevalence, and mortality of BC will be considered.

### 2.4 Setting

No restriction on clinical setting would be implied. Studies carried out at all levels of health care setting (primary, secondary, and tertiary health care) and reviews in the community will be included. Language studies published in the English language.

### 2.5 Selection process

Independent screening of the relevant titles and abstracts will be obtained with the selection criteria by the authors. The full-text articles of the eligible studies will be downloaded. Eligibility assessment will be performed independently in an unblinded standardized manner by carrying out the 1st screening of the records in parallel using a defined set of inclusion and exclusion criteria of participants and studies. Following the search, all identified citations will be collated and uploaded into citation management system (EndNote X6 Clarivate Analytics, PA) and duplicate removed. All the authors will examine abstracts, and a file will only be removed when there is a mutual agreement that it did not fulfill the selection criteria. The results of the search will be reported in full in the final systematic review and presented in a Preferred Reporting Items for Systematic Reviews and Meta-analyses (PRISMA) flow diagram. Potentially relevant studies will be retrieved in full and their citation details imported into the reference management system.

### 2.6 Assessment of risk of bias in individual studies

The risk of bias will be evaluated by the authors based on parameters such as the number of patients studied, year of publication, mode of disease diagnosis, geographical demarcation, and period of study. A predefined checklist from Dutch Cochrane using the Meta-analyses Of Observational Studies in Epidemiology (MOOSE) guidelines^[9]^ will be used to assess the quality of the studies. The reporting characteristics of the tool consist of 6 elements such as background, search strategy, methods, results, discussion, and conclusions. The reporting characteristics in the checklist are based on epidemiologic principles despite the scarcity of substantial empirical evidence in individual studies.^[10]^

We will undertake a quality component analysis to assess the quality of registry data that could be explored into 2 different domains. Firstly, we could evaluate the data in terms of research quality, in relation to the scientific process particularly the design and operational aspects of the registry, and secondly look into evidence quality of the registry data, which connects to the data/findings emanating from the research process.^[11]^

In addition, we would assess the quality of registry data using purpose, patient population, data quality, data completeness and data analysis methods. The studies will be assessed for risk of bias using the guideline formulated by Effective Health Care Program,^[12]^ and we will also use the Newcastle–Ottawa scale for the methodologic assessment of cohort studies.^[13]^

Following critical appraisal, studies that do not meet a certain quality threshold will be excluded. This decision will be based on the exclusion criteria.

### 2.7 Data extraction and management

The authors will independently evaluate the studies with the selection criteria. For missing information, corresponding authors of the studies will be contacted. Disagreements between the authors will be resolved through discussion and consultation with a 3rd reviewer. Preferred Reporting Items for Systematic review and Meta-Analysis (PRISMA) guidelines^[8]^ will be used to prepare the data extraction form using MS Excel. The bibliographic and demographic information will be collected in the data extraction form in addition to clinicoepidemiologic, clinicopathologic, and biologic characteristics of BC participants (if sufficient study information and data are identified and available). The age-standardized method by Segi standard population (Segi, 1960).^[14]^ Modified by Doll et al (1966) will be used for comparing incidence rates across the various registries.^[15,16]^

### 2.8 Data collection process

The following information will be extracted from the studies.

#### 2.8.1 Data items

1. Characteristics of the study (including author, year of publication, a geographic region within India that the study talks about, the year when the study took place, and type of research).
2. Characteristics of study methods, including International Classification of Disease (ICD) code for the anatomical site of cancer under study and number of cases/patients.
3. A statistical measure of the epidemiology of BC in India such as incidence (information on crude rate [CR] and age-adjusted rate [AAR] per 100,000 population), prevalence (1-, 3-, and 5-year), mortality (age-standardized), age-wise BC trends, and projection of burden of BC.
4. Clinicoepidemiologic characteristics of study participants (including age, study design, follow-up, setting, geographical locations, population-based, and economic status).
5. Clinicopathologic and biologic characteristics of study participants, including histologic type (lobular and ductal), lymphovascular invasion, tumor size (T1, T2, and T3), histologic grade (G1, G2, and G3), clinical stage (0–I, II, and III–IV), positive lymph node status, estrogen receptor and progesterone receptor (ER and PR) status (positive or negative), human epidermal growth factor receptor 2 (HER2), TNBC, BReast CAncer 1 and 2 (BRCA 1 and BRCA 2) (mutated and nonmutated), urokinase plasminogen activator, and plasminogen activator inhibitor 1, if sufficient study data are identified and available.^[17–20]^

#### 2.8.2 Data synthesis

The epidemiologic data source will be identified and along with trends in the analysis of age-standardized incidence; 1-, 3-, and 5-year prevalence; and age-standardized mortality of BC in India.

### 2.9 Meta-analysis

Meta-analyses on BC epidemiology in India will be performed using the software Comprehensive Meta-Analysis 3.0 for the obtained odds ratios (ORs) and 95% confidence intervals (CIs) from the included studies. Heterogeneity will be calculated using Cochrane *Q* test^[21]^ and *I*^2^ statistic.^[22]^ The variation between and within the included studies is analyzed by *I*^2^ statistic while *Q* test gives the difference between the fixed effect and the observed effect by summing up and squaring their differences. *Z*-statistic will be performed to assess heterogeneity. Publication bias will be quantified using Egger bias indicator test, Orwin^[23]^ and Classic fail-safe *N* test, Begg and Mazumdar rank collection test, Duval and Tweedie trim and fill^[24]^ calculation and inverted funnel plot.

### 2.10 Subgroup analyses and meta-regression

Subgroup analyses or meta-regression will be performed according to BC participants’ clinicoepidemiologic, clinicopathologic, biologic characteristics, and methodologic factors if sufficient studies and retrieved data are identified and available. Our research team plans to investigate specific subgroup analyses according to clinicoepidemiologic characteristics and demographic features of identified study participants. Further specific subgroup analyses will be performed based on clinicopathologic information such as the age of onset, menstruation status (pre- or postmenopausal), oral contraceptive use, operation, family cancer history, and the risk of recurrence and metastasis in different subtypes of BC in India. We will conduct specific subgroup analyses or meta-regression based on the clinicopathologic characteristics of 4 subtypes of BC: luminal A, luminal B, HER2, TNBC, and their associated prognostic factors. If sufficient BC participants’ accessory clinicopathologic information is identified, a specific subgroup analyses will be performed based on histologic type (lobular and ductal), lymphovascular invasion, tumor size (T1, T2, T3, and T4), histologic grade (G1, G2, and G3), clinical stage (0–I, II, and III–IV), lymph node status, ER and PR status (positive or negative), and BRCA 1 and BRCA 2 (mutated and nonmutated).^[17–20]^

The heterogeneity of relative contributions of the study designs, populations, and time period associations with 1 or more study key variable will be assessed using meta-regression analysis. The impact of proportional contributions of these variables individually and in combination on fitting covariables including gender distribution, methods of data collection, sample size, research quality, and sampling procedure will be calculated using meta-regression model. Tables, flowchart and figures will be plotted to depict the results in an informative and appealing manner.

### 2.11 Reporting of this review and its findings

**Figure 1:**
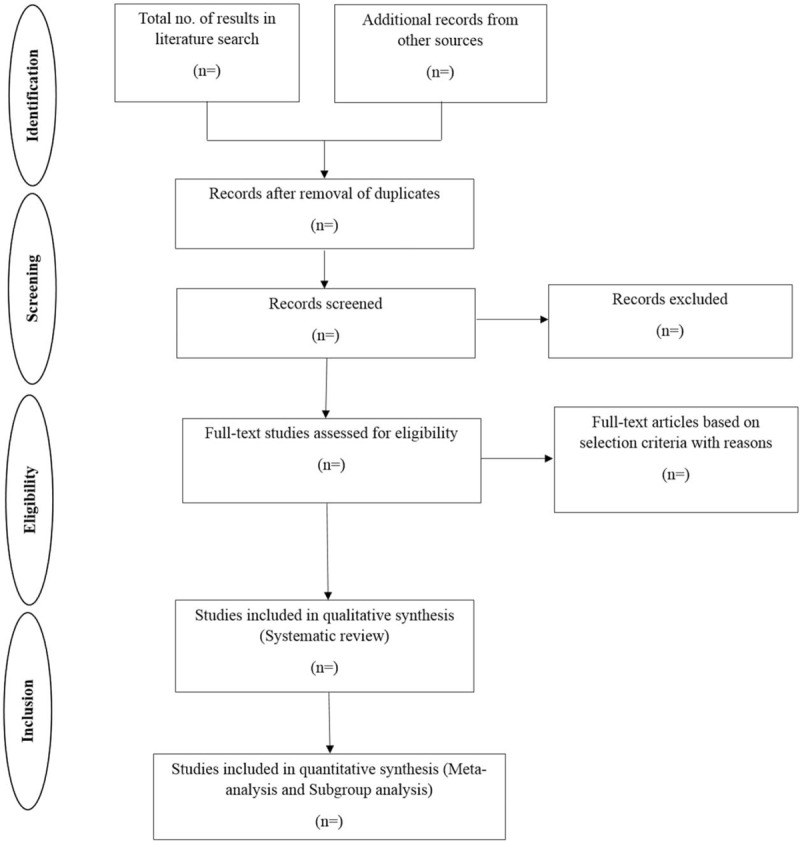
Flowchart for the systematic review.

The findings will be published as per PRISMA guidelines.^[8]^ A flowchart will be employed to outline the selection process (Fig. 1) and PRISMA-P checklist.^[25]^ The qualitative data of the included studies will be reviewed descriptively. Outputs of meta-analyses will be depicted in a forest plot. Publication bias will be represented in an inverted funnel plot. The search strategy and quality appraisal tool will be supplemented.

### 2.12 Ethics and dissemination

This study will be performed using publicly available anonymized data without involving human participants; therefore, it does not require formal human research ethical review nor approval from the human research ethics committee. We plan to disseminate our findings through publication in a peer-reviewed journal and presentation at relevant conference proceedings. In addition, we believe the results of the systematic review will have implications for policy and practice. We will prepare a policy-maker friendly summary using a validated format and disseminate through social media and email discussion groups.

### 2.13 Assessing certainty in the findings

Summary of findings (SoF) will be created to present the following information where appropriate: estimates on the incidence, prevalence and mortality of BC, estimates of OR, and a ranking of the quality of the evidence based on the risk of bias, directness, heterogeneity, precision, and risk of publication bias of the review results.

The outcomes reported in the SoF will be the incidence, prevalence, and mortality of BC in 29 states and 7 union territories of India.

### 2.14 Patient and public involvement

No patients and or public were involved in the proposed systematic review and meta-analysis study.

## Acknowledgment

The authors acknowledge the meta-analysis concepts and applications workshop manual by 46 Michael Borenstein for his guidelines on reporting meta-analysis, subgroup analysis, and publication bias (www.meta-analysis-workshops.com).

## Author contributions

RJ, SGN, and MRM contributed to the conceptualisation, study design, search strategy, protocol development and review by revising different versions. RJ, NR and KMG were involved in the supervision, ensured the absence of errors and arbitrated in case of disagreement. KB, SS, VP and DGKD engaged in the manuscript writing and analysis. All authors have read and approved the final version of the manuscript.

**Conceptualization:** Madurantakam Royam Madhav, Krishnav Biyani, Venkatesh Pandey, Shanthi Sabarimurugan, K M Gothandam, Rama Jayaraj.

**Data curation:** Madurantakam Royam Madhav, Sankaranarayanan Gomathi Nayagam, Krishnav Biyani, Venkatesh Pandey, Durai Gowtham Kamal, Shanthi Sabarimurugan, Nachimuthu Ramesh, K M Gothandam, Rama Jayaraj.

**Formal analysis:** Madurantakam Royam Madhav, Sankaranarayanan Gomathi Nayagam, Krishnav Biyani, Venkatesh Pandey, Durai Gowtham Kamal, Shanthi Sabarimurugan, Nachimuthu Ramesh, K M Gothandam, Rama Jayaraj.

**Funding acquisition:** Nachimuthu Ramesh.

**Investigation:** Madurantakam Royam Madhav, Krishnav Biyani, Venkatesh Pandey, Shanthi Sabarimurugan, Nachimuthu Ramesh, K M Gothandam, Rama Jayaraj.

**Methodology:** Madurantakam Royam Madhav, Sankaranarayanan Gomathi Nayagam, Krishnav Biyani, Venkatesh Pandey, Shanthi Sabarimurugan, Nachimuthu Ramesh, K M Gothandam, Rama Jayaraj.

**Project administration:** Madurantakam Royam Madhav, Sankaranarayanan Gomathi Nayagam, Krishnav Biyani, K M Gothandam, Rama Jayaraj.

**Resources:** Rama Jayaraj.

**Software:** Rama Jayaraj.

**Supervision:** Nachimuthu Ramesh, K M Gothandam, Rama Jayaraj.

**Validation:** Durai Gowtham Kamal, Shanthi Sabarimurugan, Rama Jayaraj.

**Writing – original draft:** Madurantakam Royam Madhav, Sankaranarayanan Gomathi Nayagam, Krishnav Biyani, Venkatesh Pandey, Durai Gowtham Kamal, Shanthi Sabarimurugan, Nachimuthu Ramesh, K M Gothandam, Rama Jayaraj.

**Writing – review & editing:** Madurantakam Royam Madhav, Sankaranarayanan Gomathi Nayagam, Krishnav Biyani, Venkatesh Pandey, Durai Gowtham Kamal, Shanthi Sabarimurugan, Nachimuthu Ramesh, K M Gothandam, Rama Jayaraj.

Rama Jayaraj orcid: 0000-0002-2179-0510.

## Supplementary Material

Supplemental Digital Content
