# Supplementary material for: Epidemiologic analysis of breast cancer incidence, prevalence, and mortality in India: Protocol for a systematic review and meta-analyses
Source: Medicine (Baltimore). 2018 Dec 28;97(52):e13680. doi: 10.1097/MD.0000000000013680 (PMC6314759; doi:10.1097/MD.0000000000013680)
Supplement: Supplemental Digital Content [file medi-97-e13680-s001.docx]

**Appendix 1 Search strategy**

| **S.No** | **Search terms** |
| --- | --- |
| 1 | “Cancer” [Topic] AND “India” [Topic] |
| 2 | “Cancer” [Topic] AND “Epidemiology” [Topic] AND “India” [Topic] |
| 3 | “Cancer” [Topic] AND “Incidence” [Topic] AND “India” [Topic] |
| 4 | “Cancer” [Topic] AND “Prevalence” [Topic] AND “India” [Topic] |
| 5 | “Cancer” [Topic] AND “Mortality” [Topic] AND “India” [Topic] |
| 6 | “Cancer” [Topic] AND “Incidence” [Topic] AND “Prevalence” [Topic] AND “Mortality” [Topic] AND “India” [Topic] |
| 7 | “Breast Cancer” [Topic] AND “India” [Topic] |
| 8 | “Breast Cancer” [Topic] AND “Epidemiology” [Topic] AND “India” Topic] |
| 9 | “Breast Cancer” [Topic] AND “Incidence” [Topic] AND “India” [Topic] |
| 10 | “Breast Cancer” [Topic] AND “Prevalence” [Topic] AND “India” [Topic] |
| 11 | “Breast Cancer” [Topic] AND “Mortality” [Topic] AND “India” [Topic] |
| 12 | “Breast Cancer” [Topic] AND “Incidence” [Topic] AND “Prevalence” [Topic] AND “Mortality” [Topic] AND “India” [Topic] |
